# Autoimmune Gastritis Complicated by Primary Biliary Cholangitis: A Report of Two Cases and Literature Review

**DOI:** 10.7759/cureus.80208

**Published:** 2025-03-07

**Authors:** Miwako Toyoda, Masataka Ichikawa, Kenji Nakamura, Hiroshi Kishikawa, Jiro Nishida

**Affiliations:** 1 Gastroenterology, Tokyo Dental College Ichikawa General Hospital, Chiba, JPN

**Keywords:** anti-thyroid antibody, autoimmune disease, autoimmune gastritis, macrocytic anemia, primary biliary cholangitis

## Abstract

Although autoimmune gastritis (AIG) is often associated with various autoimmune diseases, including type 1 diabetes and Hashimoto’s thyroiditis, it is rarely associated with primary biliary cholangitis (PBC). Herein, we present two cases in which AIG was found to be a complication during follow-up of PBC, both histologically diagnosed as AIG with positive autoantibodies and also serologically typical for AIG, including pepsinogen and gastrin. In the first case, a 60-year-old woman with PBC was suspected of having AIG based on endoscopic findings. A definitive diagnosis of AIG was established based on a positive anti-parietal cell antibody test and characteristic histologic findings. The patient exhibited a low pepsinogen I level (5.5 pg/mL; normal: >70 pg/mL) and hypergastrinemia (637 pg/mL; normal: 37-172 pg/mL). Additionally, she was diagnosed with Hashimoto’s thyroiditis, confirmed by the presence of anti-thyroid antibodies and an enlarged thyroid gland.

In the second case, a 70-year-old woman with PBC was suspected of having AIG due to the low pepsinogen I level (5.8 pg/mL). The definitive diagnosis was established based on the presence of positive anti-intrinsic factor antibodies and characteristic histologic findings. The patient also exhibited hypergastrinemia (2845 pg/mL), but no additional autoimmune diseases were observed. In reviewing past case reports, we identified seven previous reports of AIG with PBC other than our two cases. Approximately half (4/9) were macrocytic anemia, and approximately half (4/9) were positive for anti-thyroid antibodies, which is higher than that expected for conventional PBC. Because AIG should not be misdiagnosed because of the high frequency of associated malignancies, other concomitant autoimmune diseases, and vitamin B12 deficiency, it should be considered in the surveillance of PBC, especially in those with macrocytic anemia or positive antithyroid antibodies.

## Introduction

Autoimmune gastritis (AIG) is now widely understood by clinicians, and it has become clear that it is more common than previously expected [1]. Accurately diagnosing the disease is crucial because gastric cancer and neuroendocrine tumors are frequent complications [2], and the disease can trigger other autoimmune diseases [3], as well as neurological disorders and macrocytic anemia due to vitamin B12 deficiency. However, the diagnostic criteria vary, and some aspects remain ambiguous. In Japan, autoantibody seropositivity (anti-parietal cell antibody and/or anti-intrinsic antibody) and findings compatible with AIG in either endoscopic or histologic findings have been used as diagnostic criteria by clinicians [4]. However, some researchers believe that AIG cannot be diagnosed without histology. There are also cases where accurate diagnosis is difficult because of false-positive or false-negative results for autoantibodies or when AIG is not accurately suspected by histology. Thus, it is important to educate pathologists about the typical pathological findings of AIG. Pepsinogen and gastrin levels are often abnormal in AIG due to the disappearance of chief cells and hypergastrinemia induced by hypoacidity. This suggests that AIG can be predicted using a cutoff value of these serological markers [5].

As already mentioned, AIG is often complicated by other autoimmune diseases. Hashimoto’s thyroiditis is the most common autoimmune complication of AIG, but type 1 diabetes, rheumatoid arthritis, Sjögren’s syndrome, and vitiligo can also occur [3]. Primary biliary cholangitis (PBC), a chronic, autoimmune-related progressive cholestasis common among middle-aged women, also occurs frequently in combination with other autoimmune diseases, and the most common of these are rheumatoid arthritis, Raynaud’s phenomenon, and Sjögren’s syndrome [6]. A report revealed that AIG occurred in three of 111 cases (3%) in patients with PBC [7]. Furthermore, 4% of patients with PBC have pernicious anemia, most of whom are speculated to have advanced-stage AIG [8]. The prevalence of anti-intrinsic factor antibody positivity for AIG in PBC is 3.8% [9]. Therefore, the estimated incidence of AIG in PBC is approximately 3-4%. However, there are few reports regarding the clinical picture of AIG complicated with PBC.

This article presents two cases of AIG identified during follow-up for PBC and reviews previously reported cases of PBC and AIG complications. In particular, we discuss what clinical background of PBC should be considered concerning the possibility of AIG complications.

## Case presentation

Case 1

A woman in her 60s was diagnosed with asymptomatic PBC in her 50s. She was treated with ursodeoxycholic acid and monitored at our hospital’s gastroenterology department. The anti-mitochondrial M2 antibody index was 14.1 (normal, <7). At the initial examination, the liver function test results were as follows: aspartate transaminase (AST): 36 U/L (normal: 10-40 U/L); alanine transaminase (ALT): 32 U/L (normal: 5-45 U/L); alkaline phosphatase (ALP): 303 U/L (normal: 100-325 U/L); and gamma-glutamyl transpeptidase (γGTP): 78 U/L (normal: <30 U/L). γGTP levels were slightly elevated, but bilirubin levels were normal (Table 1).

**Table 1:** Laboratory data of patients with autoimmune gastritis complicated by primary biliary cholangitis AST: aspartate transaminase, ALT; alanine transaminase, ALP; alkaline phosphatase, γGTP; gamma-glutamyl transpeptidase. *; Data were obtained at the initial diagnosis of PBC. †; Data were obtained at the initial diagnosis of autoimmune gastritis.

Tests	Case 1	Case 2	Reference range
AST (U/L)*	36	35	10-40
ALT (U/L)*	32	49	5-45
ALP (U/L)*	303	195	100-325
γGTP (U/L)*	78	320	<30
Anti-mitochondrial M2 antibody index*	14.1	15	< 7
Hemoglobin (g/dL)^†^	12.2	10.8	11.5-15
Mean corpuscular volume^†^(fL)	97.2	76.3	85-102
Pepsinogen-I (ng/mL)^†^	5.5	6.4	>70
Pepsinogen-I/II ratio^†^	0.7	0.6	>3
Gastrin (pg/ml)^†^	486	637	37-172
Anti-parietal cell antibody (fold)^†^	40	<10	<10
Anti-intrisic factor antibody^†^	Negative	Positive	Negative
Anti-thyroglobulin antibody (IU/mL)^†^	71.8	0.72	<4.1
Anti-thyroid peroxidase antibody (IU/mL)^†^	2000	0.53	<5.61

Autoimmune hepatitis was ruled out, and hepatitis B virus (HBV) and C virus (HCV) markers were negative. An abdominal ultrasound was performed to investigate the cause of cholestasis, excluding biliary and infiltrative disease. PBC was diagnosed using the positive anti-mitochondrial antibody and unexplained cholestasis based on the European Association for the Study of the Liver (EASL) practical clinical guidelines.

**Figure 1:**
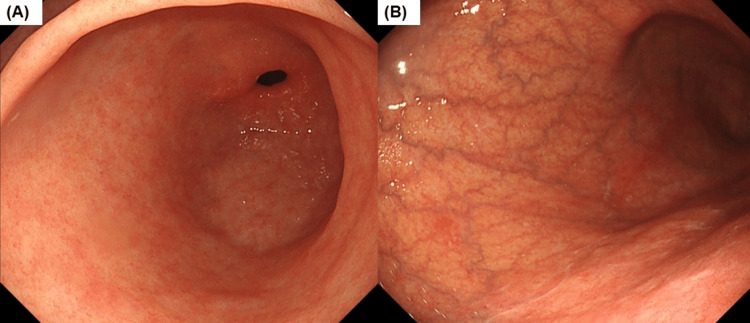
White light endoscopic image in Case 1 The atrophy of the mucosa in the antrum is mild (A), but vascular visibility and the disappearance of the gastric fold are present in the corpus (B), indicating predominant corpus atrophy.

Upper gastrointestinal endoscopy for surveillance of atrophic gastritis revealed a “reverse atrophy pattern,” in which atrophy of the corpus was more severe than that of the fundus. Thus, AIG was suspected endoscopically (Figures 1A, 1B). The anti-parietal cell antibody titer was 40-fold positive. The pepsinogen (PG) I, PG I/II ratio, and gastrin levels were 5.5 pg/mL (normal: >70 pg/mL), 0.7 (normal: >3), and 486 pg/mL (normal: 37-172 pg/mL), respectively (Table 1), suggesting advanced atrophy.

**Figure 2:**
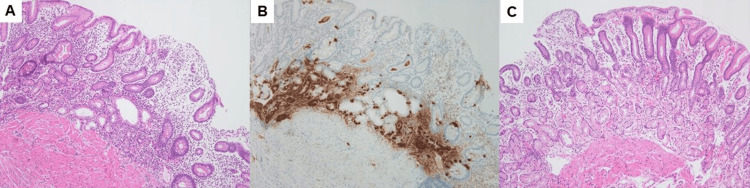
Histological findings of Case 1 Hematoxylin and eosin staining of the greater curvature of the stomach shows decreased parietal cells, intestinal metaplasia, pyloric metaplasia, and inflammatory cell infiltration, mainly in the deeper layers (X100, magnification). The ratio of gastric pits to ducts is 1:0.5 (A). Chromogranin A staining shows linear hyperplasia of enterochromaffin-like cells in some areas (X100, magnification) (B). The antrum biopsy shows preserved pyloric glands and little inflammatory cell infiltration (Hematoxylin and eosin staining; X100 magnification) (C). These findings are consistent with *H. pylori*-negative “pure AIG” in the advanced florid stage.

A biopsy of the greater curvature of the body showed a gastric pits-to-gastric-ducts ratio of 1:0.5, a significant decrease in parietal cells, and inflammatory cell infiltration, which was predominantly observed in the deeper layers (Figure 2A). Additionally, linear hyperplasia of the enterochromaffin-like (ECL) cells (Figure 2B) was observed with chromogranin A staining. However, a biopsy of the antrum showed little inflammatory cell infiltration and no intestinal metaplasia with mild atrophy (Figure 2C). Taken together, the pathological and endoscopic findings were typical of AIG without current or past *H. pylori *infection, and the patient was positive for anti-parietal cell antibodies. Therefore, we diagnosed advanced florid stage AIG based on the Japanese diagnostic criteria reported by Kamada et al. [4]. The patient had no history of eradication of *H. pylori*, the corpus-predominant atrophy and inflammation pathological findings were consistent with AIG, and the stool antigen test was negative for *H. pylori*. Therefore, we diagnosed this patient with pure AIG without past or current *H. pylori* infections [3].

We also examined the patient for other autoimmune diseases. The patient tested positive for anti-thyroglobulin (71.8 IU/mL; normal: <4.1 IU/mL) and anti-thyroid peroxidase (2000 IU/mL; normal: <5.61 IU/mL) antibodies. Ultrasonography of the neck showed an enlarged thyroid gland, and the patient was diagnosed with Hashimoto’s thyroiditis despite normal thyroid function. Finally, during follow-up for PBC, the patient was found to have a combination of AIG and Hashimoto’s thyroiditis and is currently being followed up as an outpatient.

Case 2

The patient was a woman in her 70s. She had been diagnosed with liver dysfunction due to asymptomatic PBC in her 50s. She was treated with ursodeoxycholic acid after a positive anti-mitochondrial M2 antibody test with an index of 15 (normal: <7). At the initial examination, the AST and ALT levels were 35 U/L (normal: 10-40 U/L) and 49 U/L (normal: 5-45 U/L), respectively, and the γGTP and ALP levels were 320 U/dL (normal: <30 U/L) and 195 U/dL (normal: 100-325 U/L), respectively, indicating high γGTP levels (Table 1). HBV and HCV tests were negative, and autoimmune hepatitis was ruled out. Ultrasound showed normal liver parenchyma and no gallstones, and extrahepatic cholestasis was excluded. PBC was diagnosed based on EASL clinical practice guidelines with anti-mitochondrial antibody positivity and unexplained cholestasis, as in Case 1.

**Figure 3:**
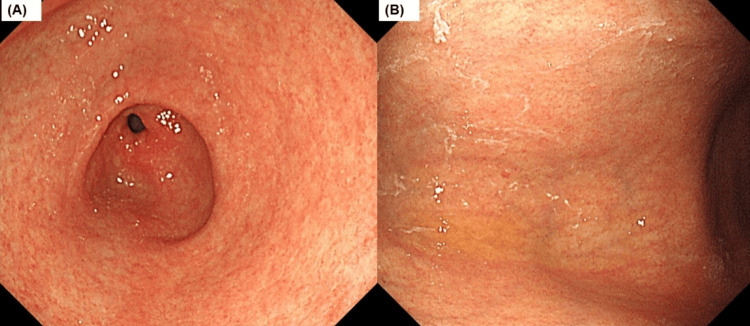
White light endoscopic image in Case 2 The mucosa in the antrum is atrophic (A), and vascular visibility and the disappearance of the gastric fold are present in the corpus (B), indicating that endoscopic examinations do not necessarily show atrophy predominantly in the corpus of the stomach.

In her 60s, the patient was diagnosed with an *H. pylori* infection based on a positive urea breath test, which was successfully eradicated. The PGI, PGI/II ratio, and gastrin levels were 5.8 pg/mL (normal: >70 pg/mL), 0.7 (normal: >3), and 637 pg/mL (normal: 40−200 pg/mL), respectively, suggesting advanced atrophy (Table 1), and AIG was suspected based on the serological findings. Endoscopy did not show typical corpus-predominant atrophy; however, visible sticky mucus was observed (Figures 3A, 3B). The patient tested negative for anti-parietal cell antibodies but positive for anti-intrinsic factor antibodies.

**Figure 4:**
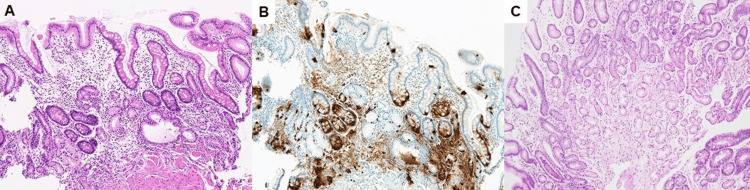
Histological findings of Case 2 Hematoxylin and eosin staining of the greater curvature of the stomach shows almost complete loss of parietal cells, significant intestinal metaplasia, pyloric metaplasia, and inflammatory cell infiltration, mainly in the deeper layers (X100, magnification). The ratio of gastric pits to ducts is 1:0.2 (A). Chromogranin A staining shows linear hyperplasia of enterochromaffin-like cells in some areas (X100, magnification) (B). The antrum biopsy shows decreased pyloric glands and mild inflammatory cell infiltration, however, the degree of atrophy is less severe than that in the corpus (hematoxylin and eosin staining; X100, magnification) (C). These histological findings are consistent with those of patients after successful eradication of *H. pylori* in advanced end-stage AIG.

Furthermore, the pathological examination of the biopsies from the corpus showed a gastric-pits-to-gastric ducts ratio of 1:0.2, with the disappearance of parietal cells, pseudopyloric metaplasia, significant intestinal metaplasia (Figure 4A), and linear ECL cell hyperplasia (Figure 4B). In the antrum, there was inflammatory cell infiltration and a decrease in the number of pyloric glands, but the degree of atrophy in the antrum was less severe than in the corpus, and these histologic findings were consistent with those of AIH with a history of *H. pylori* infection (Figure 4C). Although endoscopic findings were not typical for AIG, pathological and serological findings were compatible with AIG; thus, the patient was diagnosed with the advanced end stage of AIG with a history of *H. pylori* infection, based on the criteria by Kamada et al. [4].

Moreover, we assessed the patient for other autoimmune diseases, which revealed the presence of diabetes. However, she tested negative for anti-glutamic acid decarboxylase antibodies; thus, we ruled out type 1 diabetes. She also tested negative for anti-thyroid autoantibodies. Therefore, we diagnosed AIG complicated by PBC, and the patient is currently being monitored in an outpatient clinic.

## Discussion

We encountered two cases in which co-existing AIG was identified during follow-up for PBC. Case 1 was initially diagnosed based on typical endoscopic findings, and Case 2 was initially diagnosed using PG test results. We conducted a PubMed search to identify related cases and found seven cases in which the clinical information was evaluated among the cases of AIG co-existing with PBC. [10-16] (Table 2). Of the nine patients, including two from our report, eight were positive for either anti-parietal cell or anti-intrinsic factor antibodies and met the pathological criteria for AIG. The remaining patient tested positive for anti-intrinsic factor antibodies and showed atrophic changes predominantly in the corpus on endoscopy, although no histological evaluation was performed. Consequently, all patients met the Japanese diagnostic criteria reported by Kamada et al. [4].

Regarding the laboratory findings of these cases, macrocytic anemia (mean corpuscular volume >100 fL and hemoglobin level <11.4 g/dL in females) was observed in four cases (44%). Anti-thyroid antibody positivity was also observed in four cases (44%). The reported rate of macrocytic anemia in AIG ranges from 39.1% to 50% [17], and the rate of anti-thyroid antibody positivity ranges from 38% to 53% [16], similar to cases with AIG co-existing with PBC. The gastrin levels of the nine previously reported cases (475-2955 pg/mL) are similar to those reported in an AIG case series (median: 1310 pg/mL [5] and mean: 2845 pg/mL [17]). Therefore, it is thought that most of the reported cases of AIG complicated with PBC showed typical laboratory findings similar to advanced florid-phase AIG.

However, as approximately half of the cases showed macrocytic anemia and positive anti-thyroid antibodies, our findings were unexpectedly higher in patients with AIG complicated with PBC than in many patients with PBC alone, which is an important finding. In other words, pernicious anemia and Hashimoto’s thyroiditis co-occur with PBC infrequently and have been reported in only 4% and 9.1% of cases, respectively [18]. Therefore, AIG should be strongly suspected if macrocytic anemia or Hashimoto’s thyroiditis is observed in PBC.

**Table 2:** The clinical characteristics of reported autoimmune gastritis cases complicated by primary biliary cholangitis F; Female, MCV; Mean corpucular volome, PA; Pernicious anemia, APCA; Anti-parietal cell antibody, AIF; Anti-intrinsic factor antibody, AMA; Anti-mitochondrial M2 antibody,

Author	Sex	Age (years)	Hemoglobin (g/dL)	MCV (fL)	PA	Scheuer’s classification of primary biliary cholangitis	Anti-thyroid antibody	APCA	AIF	AMA	Histology compatible with autoimmune gastritis	Gastrin (pg/mL)
Aoyama H et al [10]	F	68	6.4	136	+	NA	+	+	+	+	+	1100
Takahashi et al [11]	F	52	12.9	NA	-	stage II	-	-	+	-	NA	NA
Wakabayashi et al [12]	F	45	5	63.7	-	stage II	+	NA	+	+	+	2955
Dohmen et al [13]	F	72	7.3	132	+	NA	-	+	+	+	+	2500
Jazia et al [14]	F	68	10	124	+	NA	-	+	-	+	+	NA
Mork et al [15]	F	64	NA	NA	-	NA	+	+	NA	+	+	2560
Chung et al [16]	F	46	10.5	114.8	+	stage II	-	+	NA	+	+	NA
Present study	F	60's	12.2	97.2	-	NA	+	+	-	+	+	486
	F	70's	10.8	76.3	-	NA	-	-	+	+	+	637

Some patients with PBC present with apparent portal hypertension despite laboratory data that does not suggest liver cirrhosis (e.g., low platelet counts) [19]. Therefore, it is necessary to perform regular upper gastrointestinal endoscopy to check for esophageal varices, which can lead to fatal complications when managing PBC. Most patients with PBC regularly undergo upper gastrointestinal endoscopy; thus, there are several chances to detect AIG. However, endoscopists who are unaware of the characteristic findings can misdiagnose AIG. It should be noted that during upper gastrointestinal endoscopy in a patient with PBC, characteristic findings for AIG, such as corpus-predominant atrophy, a sticky adherent mucus, a remnant oxyntic mucosa, and characteristic hyperplastic polyps, should not be overlooked. If in doubt, checking for autoantibodies, such as anti-parietal cell antibodies, as well as the pathological findings, including the disappearance of parietal cells or enterochromaffin-like (ECL) cell hyperplasia based on chromogranin A staining, should be performed to evaluate for AIG. The differential diagnosis is *H. pylori* gastritis with advanced atrophy. *H. pylori* gastritis is diagnosed by antrum-predominant atrophy, which is different from that of AIG, which shows corpus-predominant atrophy. In addition, some hypothesize that there are cases in which findings not normally seen in PBC are observed, such as iron deficiency anemia (seen in early AIG) and peripheral neuropathy caused by vitamin B12 deficiency; these observations could be due to a lack of intrinsic factors associated with parietal cell destruction. In such cases, AIG should also be suspected, and examinations for the same autoantibodies and pathologies described above should be performed.

A prior report suggested a possible link between PBC and AIG via *H. pylori *infection [20], but this has not been definitively proven. However, in four of the nine cases we reviewed, PBC, AIG, and anti-thyroid antibodies were all positive, suggesting that some autoimmune mechanisms at work are not coincidental.

## Conclusions

An accurate diagnosis of AIG is important in daily clinical practice because of the high incidence of 1) malignancies, 2) other systemic autoimmune diseases, and 3) vitamin B12 deficiency. Here, we presented two cases of AIG associated with PBC. After reviewing previous case reports, we suggest that the possibility of AIG complicating PBC, which has characteristic findings, should be considered. Considering that about half of the cases of PBC with AIG exhibited pernicious anemia or were positive for anti-thyroid autoantibodies, we suggest considering the possibility of AIG in patients with such symptoms, especially those with PBC. Even in the absence of macrocytic anemia or thyroid autoantibodies, attention should be paid to corpus-predominant atrophy during endoscopic examinations of patients with PBC, and autoantibody measurements and pathological examinations should be performed to ensure a thorough evaluation.
